# Focusing on discoidin domain receptors in premalignant and malignant liver diseases

**DOI:** 10.3389/fonc.2023.1123638

**Published:** 2023-03-15

**Authors:** Hang Gong, Hui-Mei Xu, De-Kui Zhang

**Affiliations:** Department of Gastroenterology, Lanzhou University Second Hospital, Lanzhou, Gansu, China

**Keywords:** discoidin domain receptors, liver, cancer, extracellular matrix, metastasis, therapy

## Abstract

Discoidin domain receptors (DDRs) are receptor tyrosine kinases on the membrane surface that bind to extracellular collagens, but they are rarely expressed in normal liver tissues. Recent studies have demonstrated that DDRs participate in and influence the processes underlying premalignant and malignant liver diseases. A brief overview of the potential roles of DDR1 and DDR2 in premalignant and malignant liver diseases is presented. DDR1 has proinflammatory and profibrotic benefits and promotes the invasion, migration and liver metastasis of tumour cells. However, DDR2 may play a pathogenic role in early-stage liver injury (prefibrotic stage) and a different role in chronic liver fibrosis and in metastatic liver cancer. These views are critically significant and first described in detail in this review. The main purpose of this review was to describe how DDRs act in premalignant and malignant liver diseases and their potential mechanisms through an in-depth summary of preclinical *in vitro* and *in vivo* studies. Our work aims to provide new ideas for cancer treatment and accelerate translation from bench to bedside.

## 1 Background

**Figure 1:**
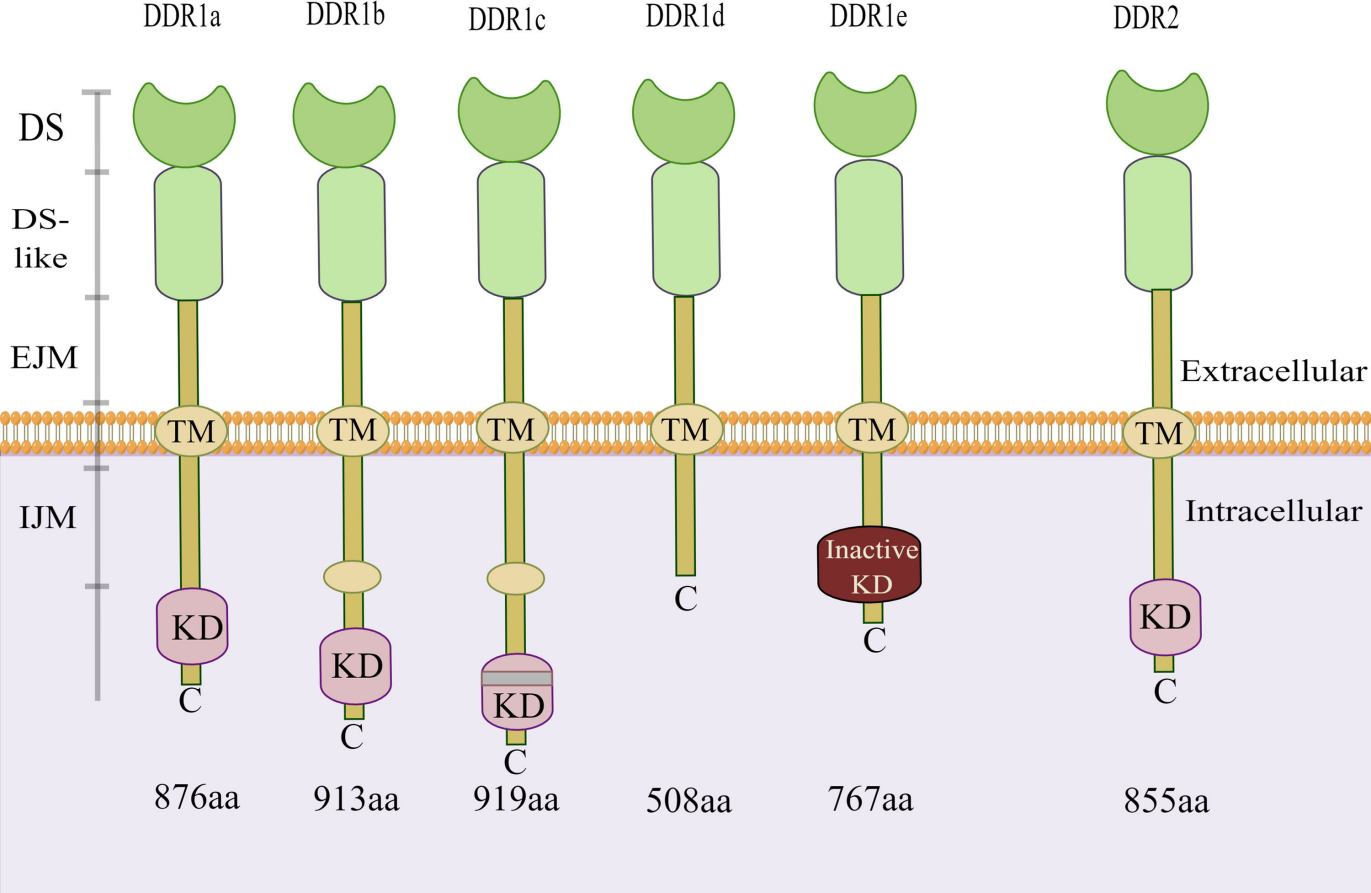
Structure of DDR1 and DDR2. There are five isoforms of DDR1. Among them, the kinase activity is present in the DDR1a, DDR1b and DDR1c coding sequences, but not in the DDR1d or DDR1e. However, only one single isoform of DDR2 exists. DDR, discoidin domain receptor; DS, discoidin domain; EJM, extracellular juxta-membrane; IJM, intracellular juxta-membrane; KD, kinase domain; TM, transmembrane domain. (The figure is drawn by Figdraw).

Discoidin domain receptors (DDRs) are novel receptor tyrosine kinases (RTKs) discovered by Johnson et al. in 1993 (1). As a RTK family members, DDRs also include two subfamily members: discoidin domain receptor 1 (DDR1) and discoidin domain receptor 2 (DDR2) (2). Similar to other RTK family members, the structure of DDRs includes an extracellular region, a transmembrane (TM) domain, and an intracellular kinase region. Unlike other members of the RTK family, the extracellular regions of DDRs contain a globular discoidin (DS) domain of 155 amino acids, a discoidin-like (DS-like) domain and a long extracellular juxtamembrane (JM) region between the DS domain and the TM domain (3, 4). The JM segment is responsible for transmitting extracellular signals to the intracellular tyrosine kinase region (5). Both of these molecular structure features indicate that DDRs can bind to their ligands through an unusual transmembrane mechanism and play a vital role in cell biology. Based on the alternative splicing of intracellular kinase mRNA, there are five subtypes of DDR1: a, b, c, d, and e. However, only one single DDR2 subtype exists. There is kinase activity in the DDR1a, DDR1b and DDR1c coding sequences but not in the DDR1d or DDR1e coding sequences due to the deletions of exon 11 and exon 12, respectively (**Figure 1**) (6, 7). Notably, in addition to their role in collagen synthesis and degradation, DDRs promote tumour cell adhesion, migration, and differentiation and the release of inflammatory factors (8, 9).

However, the underlying mechanisms of DDRs in premalignant and malignant liver disease remain obscure. In this review, the characteristics of the structure, activation and phosphotyrosine-based interactions of DDRs are briefly introduced, and the molecular mechanism of DDRs in the liver are systematically discussed, with the aim of promoting the development of DDR-targeted therapies and new cancer treatment methods.

## 2 Activation mechanism of DDRs

Most RTKs are activated by phosphorylated tyrosine residues within seconds of ligand binding, followed by a rapid decline in activity due to dephosphorylation or internalization and degradation of the receptor/ligand (10). Collagen, a key constituent of the extracellular matrix, is the specific ligand of DDRs, which are not activated by soluble peptide-like growth factors. The DS domain of DDRs binds with collagen (11). Unlike the signal transduction of other typical RTKs with acute and rapid responses, the binding of collagen to DDRs is slow and continuous (4). However, only collagen with a natural triple helical structure, but not heat-denatured collagen, can bind to DDRs (11, 12). The DS domain of DDR1 and DDR2 recognizes the specific amino acid sequence motif GVMGFO (O, hydroxyproline) on fibrillar collagen types I, II and III (13, 14). Collagen of the basement membrane (type IV) only activates DDR1, whereas collagens of types V and X only interact with DDR2 (15–17).

**Figure 2:**
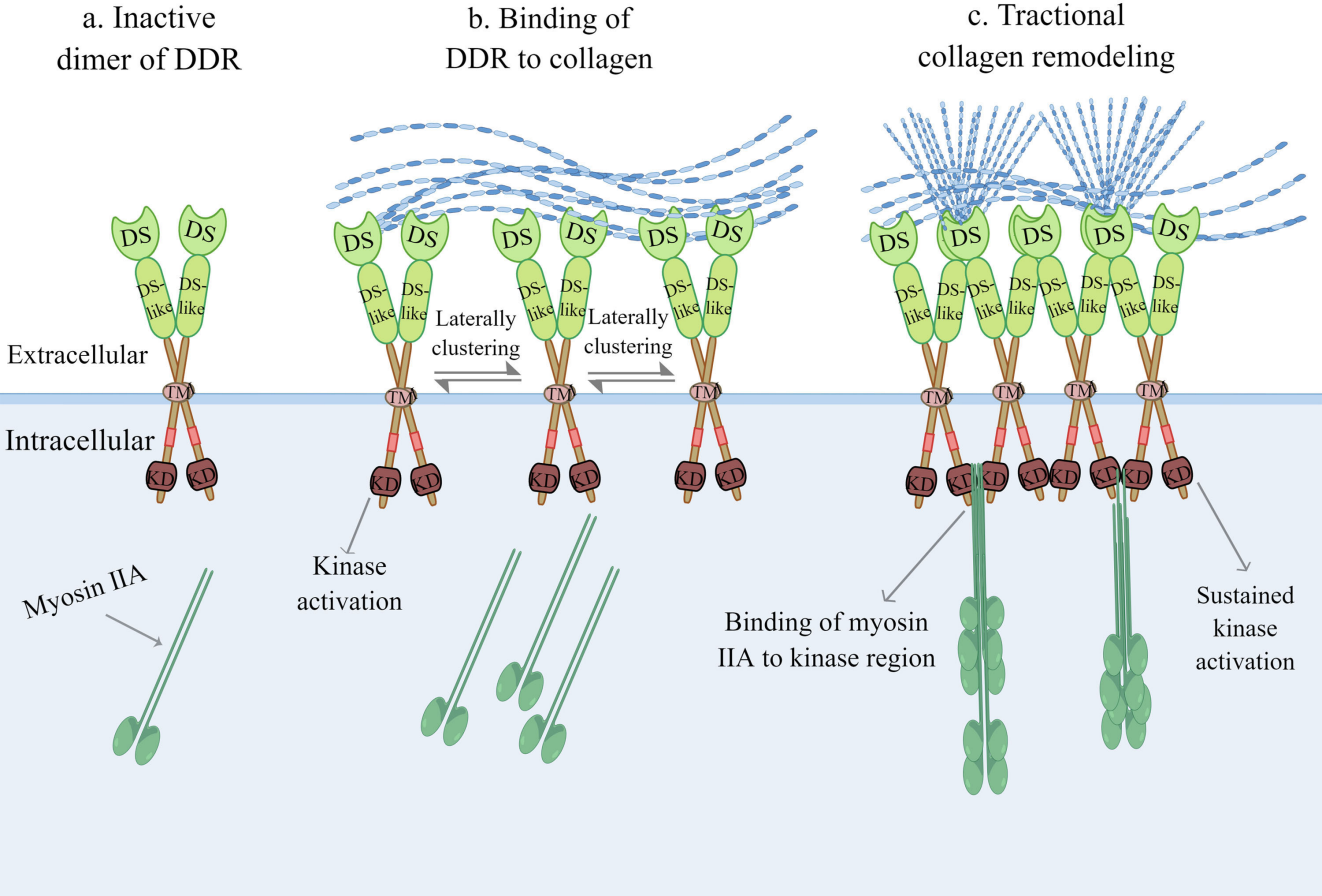
Collagen-induced DDR activation. **(A)** Inactive dimer of DDR without binding to collagen. **(B)** The activation of DDR and the lateral clusters between DDR proteins are a reciprocal causation. The lateral clusters reinforce the binding of DDR to collagen, meanwhile the increasing lateral clustering of dimers are the result of collagen binding. **(C)** Upon kinase domain activation, DDR regulates adhesion and traction force on collagen by binding to myosin IIA, which condenses collagen fibrils into more denser alignment. DDR, discoidin domain receptor; DS, discoidin domain; KD, kinase domain; TM, transmembrane domain. (The figure is drawn by Figdraw).

Although their single DS domain contains sites for binding to collagen, DDRs require dimerization of the DS domain to bind collagen with high affinity (11). Strikingly, DDRs develop independent and stable dimers mediated by one leucine-based sequence motif in the transmembrane domains in the absence of ligand recognition, which distinguishes them from other RTKs (18). Furthermore, mutation of cysteine residues in the extracellular JM region in DDRs results in independent and covalent dimers, which occur during biosynthesis, according to the study authors (19). The collagen binding region of charged residues surrounded by the hydrophobic layer in the three surface-exposed loops of the DS domain is activated by forming lateral clusters after binding to collagen (**Figure 2**) (11, 20). In other words, the activation of DDRs and the lateral clusters between DDR proteins are reciprocal causations. The lateral clusters reinforce the binding of DDRs to collagen and induce the activation of DDRs, while the lateral clusters leading to trans-phosphorylation between dimers are the result of collagen binding (18, 21). DDRs regulate adhesion and traction force on collagen by binding to myosin IIA, which condenses collagen fibrils into a denser alignment (22, 23). Activation of the DDR kinase domain enhances the association of the kinase domain with myosin IIA filaments, optimizing myosin-dependent contractile force delivery to collagen fibrils (21). The magnitude of DDR activation is proportional to collagen contractile forces (21). Upon binding of DDRs to collagen, autophosphorylation of the intracellular JM region and tyrosine residues of the intracellular kinase region in DDRs occurs (10). This phosphorylation recruits intracellular signalling protein complexes, such as Src Homology-23 (SH2/3) and the phosphotyrosine binding (PTB) domain, for assembling and transmitting receptor signals that play a cellular signal transducing function (11). Moreover, there is a non-canonical but functional crosstalk between insulin/insulin-like growth factor system (IIGFS) and DDRs, which was recently discovered apart from canonical collagen-dependent DDR activation. Interestingly, DDR1 functionally interacts with IIGFS better than DDR2 (24). As a complex network, IIGFS is constituted of transmembrane receptors, corresponding ligands, and binding proteins (24). Insulin-like growth factor 1 receptor (IGF1R) and insulin receptor (IR)-A, which are members of transmembrane receptors of IIGFS, and their common ligands consisting of insulin-like growth factors (IGF1 and IGF2) and insulin, are involved in the crosstalk with DDR1 (24). Upon stimulation by cognate ligands, IGF1R or IR-A physically interacts with DDR1 to induce rapid and sustained DDR1 phosphorylation, independent of the binding capacity of DDR1 to collagen (25). IIGFS not only stimulates DDR1 phosphorylation but also up-regulates DDR1 protein level to some extent *via* the activation of phosphoinositide 3-kinase (PI3K)/protein kinase B (Akt) cascade and further inhibition of downstream miR-199a-5p, a negative regulator of DDR1 (26).

## 3 Phosphotyrosine-based interactions of DDRs

**Figure 3:**
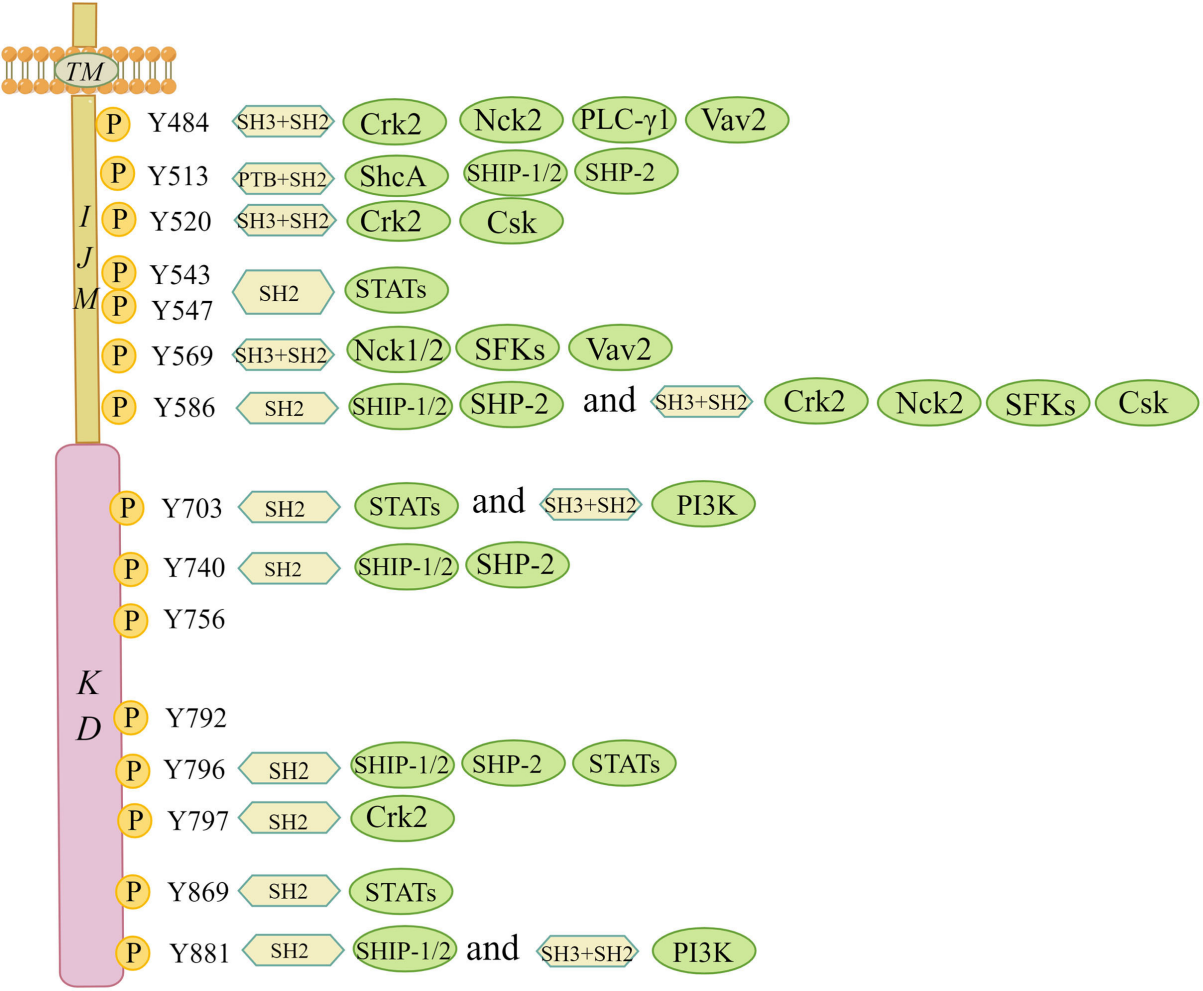
Interaction map of phosphotyrosine-based interactions of DDR1b isoform. This figure summarizes the tyrosine residue sites and corresponding interactors in the intracellular JM and kinase regions based on the study by Lemeer S et al. All phosphotyrosine interactors must rely on their SH2/3 and PTB domains in order to combine with the corresponding docking sites of tyrosine residues. Csk, C-terminal Src kinase; Crk2, adaptor protein Crk2; Nck1/2, adaptor protein Nck1/2; PI3K, phosphoinositide 3 kinase; PTB, phosphotyrosine-binding domain; PLC-γ1, phospholipase C γ1; SH2, Src homology 2 domain; SH3, Src homology 3 domain; ShcA, SH2-containing transforming protein A; SFKs, Src family tyrosine kinases; SHP-2, SH2 containing protein tyrosine phosphatase 2; SHIP-1/2, SH2-containing inositol polyphosphate 5- phosphatase 1/2; STATs, signal transducer and activator of transcriptions; TM, transmembrane domain; Vav2, guanine nucleotide exchange factor Vav2. (The figure is drawn by Figdraw).

Upon ligand activation, distinct tyrosine residues in the intracellular JM and kinase regions are phosphorylated and act as docking sites for SH2/3 or PTB, which can serve as signal adaptors to assemble intracellular signalling molecules (**Figure 3**) (10). DDR1a has 13 tyrosine residues in the cytosolic JM and kinase regions, while both DDR1b and DDR1c have 15, and DDR2 has 14. It is beyond the scope of this paper to go into their tyrosine residues, given that both DDR1d and DDR1e, with an incomplete JM domain or the absence of an entire kinase domain, are nonfunctional truncated proteins (27). Interactors of DDRs localized on the phosphotyrosine residues of the receptor have been identified. Lemeer S et al. (28) outlined the tyrosine residue sites and corresponding interactors in the intracellular JM and kinase regions based on phosphopeptide affinity purifications in human placenta. For example, Crk2 (Tyr520), ShcA (Tyr513), C-terminal Src kinase (Csk) (Tyr520), STATs (Tyr543 and Tyr 547), Nck1/2 (Tyr569), phosphoinositide 3-kinase (PI3K) (Tyr703) and other phosphotyrosine interactors containing SH2/3 and PTB domains were detected to point to the docking sites of corresponding phosphotyrosine residues in the DDR1 receptor to transduce early signals from the receptor to the downstream cascade (28). Given that DDR1a lacks Tyr513 and Tyr520 docking sites compared with DDR1b/c, adaptor molecules, such as ShcA and Csk, associated with specific docking sites cannot bind to DDR1a (12). Regarding DDR2, Iwai LK et al. (29) identified two new docking sites in the DDR2 kinase domain, namely, Tyr684 and Tyr813. Interestingly, the docking site of Tyr481 in the JM region was shown to be constitutively phosphorylated, and Tyr471 was found to be a docking point for the adaptor ShcA (29, 30). Moreover, novel adaptor molecules, such as Grb2 and EphA2, were confirmed using a DDR1b immunoprecipitation assay (28). In addition to known adaptors with well-defined functions, other new adapters appear to involve RasGAP and VAV2/3. However, whether the new adaptor molecules bind to collagen-dependent activated receptors and generate signalling effects following binding remains to be seen.

## 4 DDRs as potential therapeutic targets in premalignant and malignant liver diseases

Several cell types express DDR1, mainly epithelial cells and smooth muscle cells, as well as other types of cells, such as the epithelium of the large intestine, lung and breast cells, adrenal cortical cells, pancreatic ducts, and macrophages (31, 32). DDR2 is mainly expressed in connective tissues of mesenchymal origin, including in cartilage and smooth muscle cells, fibroblasts and myofibroblasts, but also in skeletal muscle, kidney, myocardium, and lung cells (2, 23, 33, 34). Both DDR1 and DDR2 play important roles in the process of embryonic development. DDR1 is mainly involved in organ development and maturation, while DDR2 is involved in bone growth in humans (35). The physiological effects of DDRs do not persist into human adulthood, even though they play important physiological roles during embryonic development (27). Their physiological effects are not carefully elaborated here. The pathological functions of DDRs in premalignant and malignant liver diseases are described in the following sections (**Table 1**).

**Table 1:** The list of animal models/subjects, cell lines and effects of DDRs in premalignant and malignant liver diseases.

Liver diseases	Study/ Years of Publication	Animal models/subjects	Cell lines	Effects
Pre-malignant liver diseases	Luo Z et al., 2013 (36)	Male Sprague-Dawley rats given intragastric alcohol infusion	HSC T6 cells	Acetaldehyde increases the protein level of DDR2 and MMP2 *in vitro* and *in vivo*. The silencing of DDR2 reduces MMP2 level, HSC proliferation, the synthesis of collagen I and TIMP-1 mRNA, and enhances necrosis rate and apoptosis of HSC, while also alleviating liver injury and collagen deposition in the alcohol-induced model
	Zhang Y et al., 2021 (37)	Male C57Bl/6 mice as a LF model *via* bile duct ligation and carbon tetrachloride (CCl_4_) treatment; patients with liver fibrosis or cirrhosis	HL-7702 cells	Collagen I stimulated N-terminal DDR1 shedding with a time- and concentration-dependent characteristic. Collagen I deposition and serum N-terminal DDR1 levels are proportional to the degree of liver fibrosis. The serum N-terminal DDR1 level may become a serological marker for the diagnosis of LF
	Zhang XH et al., 2010 (38)	Male Wistar rats as a LF model given intragastric alcohol infusion	NA	The DDR2 expression level was proportional to the degrees of fibrosis. expression of DDR2 mRNA was positively related to the levels of collagen type I, III, and IV, HA, laminin, and procollagen II
	Creeden JF et al., 2022 (39)	Mice administered CCl_4_ as a LF model; humans with fibrosis or cirrhosis	NA	DDR1 mRNA expression was significantly increased in cirrhosis/fibrosis humans. The phosphorylation of DDR1 has more obvious immunostaining in livers of human cirrhosis and chronic CCl_4_-treated mice
	Song S et al., 2011 (40)	Patients with end-stage Child-Pugh class C cirrhosis from HCV, AIH and PBC	Human HSC LX2 and HCC cell line Huh7	DDR1a mRNA expression is significant increased in cirrhotic livers with PBC and HCV. DDR1 protein is strongly expressed in cirrhotic liver parenchymal cells and bile ductule epithelial cells. The ectodomain of DDR1 is shed more easily in cirrhotic liver than in the non-diseased liver. Huh7 cells overexpressing DDR1 exhibit enhanced specific adhesion to collagen type I and contribute to MMP activation
	Mao TK et al., 2002 (41)	End-stage cirrhotic patients from PBC, PSC, ALD and HCV	NA	DDR2 mRNA is markedly up-regulated in cirrhotic livers. DDR2 mRNA expression is detected in all small bile duct epithelial cells and fibroblasts/stromal cells within fibrotic regions from PBC or PSC, but not the large bile ducts. However, DDR2 mRNA is confined to fibrotic lesions, but not the entire biliary epithelium in cirrhotic livers from ALD or HCV
	Shackel NA et al., 2002 (42)	End-stage cirrhotic patients from HCV and AIH	NA	The gene expression of DDR1 was up-regulated in HCV-associated cirrhosis
	Olaso E et al., 2011 (43)	Male DDR2^+/+^ and DDR2^-/-^ mice as a LF model *via* CCl_4_	HSCs and liver macrophages from DDR2^+/+^ and DDR2^-/-^ mice	Compared with DDR2^+/+^ livers, chronic CCl_4_ treatment increases DDR2^-/-^ liver fibrosis and ECM remodeling. The expression of DDR2 in HSCs is enhanced after CCl_4_ treatment. Collagen type I, MMP-2, and MMP-13 mRNA expression are reduced significantly in HSCs from chronic CCl4-injured DDR2^-/-^ livers. The migration and proliferation of HSCs from chronically injured DDR2^-/-^ livers were enhanced, and the matrix degradation was reduced. Macrophages from fibrotic DDR2^-/-^ livers show stronger chemoattractive activity toward DDR2^-/-^ HSCs
Primary HCC	Shen Q et al., 2010 (44)	HCC patients who underwent liver resection	HCC cell lines Huh7, HepG2, Hep3B, SK-HEP-1 and SNU-182	In human HCC tissues, the expression of miR-199a-5p decreases, and DDR1 increases. In HepG2 and SNU182 cell lines, DDR1 expression was significantly higher than in primary human hepatocytes. Transfection of miR-199a-5p that acts directly on DDR1 could inhibit the invasion of HepG2
	Pan Y et al., 2022 (45)	Male BALB/c (nu/nu) mice; HCC patients who underwent liver resection	Human fetal liver cell line HL-7702; HCC cell lines HepG2, Hep3B, Huh7, SK-Hep1, HLF, HLE, HCCLM3, PLC/PRF/5, 97H and Bel7402	High DDR1 expression is associated with poor prognosis in HCC patients. The expression level of DDR1 is positively correlated with that of SLC1A5. DDR1 enhances the proliferation of HCC cells by the SLC1A5-mediated mTORC1 signaling pathway and induces a G1-S phase transition
	Romayor I et al., 2020 (46)	NA	Human LX2 cell; HCC cell line SK-HEP	The mRNA expression of DDR1 in SK-HEP is similar to that in the LX2 cell. Down-regulation of DDR1 decreases the phosphorylation of AKT and ERK, inhibits the expression of ICAM1, Ki67, VCAM1, and MMP9, reduces the adhesion and migration of HCC cells to collagen type I
	Ezzoukhry Z et al., 2016 (47)	NA	HCC cell lines Huh7, Hep3B, SNU398	TGF-β1 with a higher level in HCC patients induces DDR1 overexpression in a Smad4-dependent way. DDR1 silencing of HCC cells reduces the ability to type I collagen-induced linear invadosomes
	Lee JH et al., 2018 (48)	NA	HepG2 cells	Complement protein C1q induces migration and invasion of HepG2 cells by the activation and upregulation of DDR1 as well as MAPK and PI3K/Akt pathways
	Park JW et al., 2015 (49)	BALB/c-nu mice; HCC patients who underwent liver resection	Mouse embryonic fibroblast NIH3T3 cell; HCC cell lines Hep3B, SNU387, SNU182, SNU423, SNU449, and PLC/PRF5	DDR2 mRNA is significantly expressed in all HCC cell lines and human HCC tissues. DDR2 siRNA significantly reduces cell migration and invasion
	Lee NO et al., 2012 (50)	NA	HCC cell lines Hep3B, SNU387, SNU182, SNU423, SNU449, and PLC/PRF5	The expression of DDR2 mRNA is higher in HCC cells. DDR2 siRNA reduces VEGF expression in HCC cell lines under hypoxia
	Jian ZX et al., 2012 (51)	HCC patients who underwent radical hepatectomy	NA	DDR1 expression is significantly increased in the HCC patients with early recurrence compared with the non-early recurrence patients
	Xu M et al., 2021 (52)	HCC patients who underwent curative resection	NA	The level of serum DDR1 in HCC patients is significantly higher than that in chronic hepatitis and healthy individuals. The high expression of DDR1 is significantly correlated with the rate of tumor recurrence or distant metastasis and has a higher possibility of microvascular invasion and circulating tumor cells
Metastatic liver cancer	Romayor I et al., 2021 (53)	BALB/c male mice; patients with hepatic metastases from CRC	CRC cell lines human HT29 cells, murine C26 and MCA38 cells; LSECs, KCs, and HSCs from liver samples	DDR1 is highly expressed in metastatic hepatic tissue from CRC and is associated with poor prognosis. In the three CRC cell lines, the functional DDR1 (phosphorylated DDR1) increased by 1.5-fold compared with those in basal conditions. LSECs- and HSCs-derived secretomes increase DDR1 phosphorylation and MMP2 secretion in CRC cells. Silencing of DDR1 in C26 cells significantly reduces the expression of collagen and MMP2, migratory ability, and metastatic growth in the liver
	Chen LY et al., 2019 (54)	A male C57BL/6J mouse *via* submucosal injection of CMT-93 cells into the distal posterior rectum; patients with CRC	CRC cell lines human HCT116, SW480, RKO, LoVo, DLD-1 and HT-29	The expression of circ-NSD2 in liver metastases of a mouse model is higher than that in its paired primary colon tumors and non-cancerous organs. The overexpression of circ-NSD2 enhances migration and invasion of HCT116 *in vitro*. Silencing of circ-NSD2 reduces migration and invasion of HCT116 and RKO cells *in vitro*. Circ-NSD2 may up-regulate DDR1, JAG1, and their downstream signals by targeting miR-199b-5p to inhibit the growth and metastasis of CRC
	Badiola I et al., 2012 (55)	Male DDR2^+/+^ and DDR2^-/-^ mice *via* intrasplenic injection of MCA38 colon carcinoma cells	HSCs and LSECs from DDR2^+/+^ and DDR2^-/-^ mice	The metastasis size, myofibroblast density, recruitment of angiogenic LSECs, and Ki67-expressing cell fraction in DDR2^-/-^ hepatic metastases are significantly higher than those in DDR2^+/+^ hepatic metastases. DDR2^-/-^ HSCs with or without the activation by tumor significantly increase MCA38 cancer cell migration and adhesion to LSEC. DDR2 deficiency reduces gene expression of IL-18 and insulin-like growth factor-I, and increases gene expression of prometastatic factors IL-10, TGF-β, VEGF
	Badiola I et al., 2011 (56)	C57BL/6J-Hfn11 nude mice *via* intrasplenic injection of A375 cells	Human melanoma A375 cell, colon carcinoma HT29 cell, liver carcinoma SK-HEP cell, hepatic stellate cell LX2	Silencing of DDR2 in A375 cells can reduce the liver tumor metastatic capacity of nude mice and the activity of MMP2 and MMP9, and attenuate proliferation and migration capacity *in vitro*
	Yuge R et al., 2018 (57)	Female athymic BALB/c nude mice *via* injection of Hank’s balanced salt solution into the spleen; patients with GC who underwent surgical resection	Human GC cell lines MKN1, MKN45, MKN74, HSC39, TMK1, KKLS and KATO-III	DDR1 was upregulated in GC cell lines. The silencing of DDR1 reduces GC cell proliferation, migration, invasion, and metastasis, as well as tumor microvessel area. DDR1 is associated with poor prognosis

ALD, alcoholic liver disease; Akt, protein kinase B; AIH, autoimmune hepatitis; ALD, alcoholic liver disease; CRC, colorectal cancer; DDR, discoidin domain receptor; ECM, extracellular matrix; ERK, extracellular signal-regulated kinase; GC, gastric cancer; HCV, hepatitis C virus; HA, hyaluronic acid; HCC, hepatocellular carcinoma; HSC, hepatic stellate cell; IL, interleukin; ICAM1, intercellular adhesion molecule 1; JAG1, Jagged1; KC, Kupffer cell; LF, liver fibrosis; LSEC, liver sinusoidal endothelium cell; MHC, metastatic hepatic carcinoma; MAPK, mitogen-activated protein kinase; MMP, matrix metalloprotease; NA, not applicable; PI3K, phosphoinositide 3-kinase; PBC, primary biliary cirrhosis; PSC, primary sclerosing cholangitis; SMA, smooth muscle actin; SLC1A5, solute carrier family 1 member 5; TGF-β1, transforming growth factor-β1; TIMP, tissue inhibitor of metalloproteinase; VCAM1, vascular cell adhesion molecule 1; VEGF, vascular endothelial growth factor.

### 4.1 Premalignant liver diseases

The role of DDRs in the fibrosis stage of premalignant liver diseases appears to be complex, especially that of DDR2, which may exert diverse effects on the liver fibrosis stage. Overall, a positive correlation exists between the mRNA and protein expression levels of DDRs and the degree of liver fibrosis. Fibrotic livers, especially cirrhotic livers, express much higher levels of DDRs than nondiseased livers (37–42). The activation of cells that produce extracellular matrix (ECM) is an important step in tissue remodelling during liver fibrosis (58). During liver injury (prefibrotic stage), myofibroblasts derived from hepatic stellate cell (HSC) activation mainly produce collagen types I and III deposited into the nascent matrix (59). Lateral clusters of DDR proteins and enhanced binding of DDRs to collagen mediate tractional collagen remodelling, which is essential for mechanical compression and reorganization of collagen in the ECM during fibrosis (21). DDRs can also regulate cell adhesion, migration, differentiation and proliferation, depending on collagen quantity and configuration in the ECM. Song S et al. (40) showed that DDR1 overexpression enhanced hepatocyte adhesion to collagen type I and contributed to matrix metalloprotease (MMP) activation *in vitro*. Results from *in vitro* experiments suggested that DDR2 regulates the bioactivation of HSCs and the secretion of MMPs and promotes the development of fibrosis in early liver disease (36, 43). However, DDR2 may play a protective role in chronic and persistent liver fibrosis. Compared to DDR1-/- mice that were protected from chronic lung or kidney fibrosis, chronic liver fibrosis worsened in DDR2-/- mice (43, 60, 61). In disease progression, the balance between MMP2 and tissue inhibitor of metalloproteinase (TIMP) may be inclined toward MMP2. The activity of MMP2 is inhibited by TIMP in the early stage of liver injury, despite DDR2 promotion of MMP2 secretion. However, MMP2 performs limited collagen degradation, triggering proliferation, activation, and collagen production by more HSCs (62). In chronic liver fibrosis, DDR2-mediated MMP2 plays a more prominent role in the degradation of hepatic ECM than in the activation and proliferation of HSCs due to the decreased TIMP expression (62).

### 4.2 Primary hepatocellular carcinoma

DDRs play important roles in various cancer types, such as lung, gastric, liver, breast, brain and other cancers (63). DDR1 regulates HCC invasion ability, likely through MMP2 and MMP9 in the ECM (64). A study by Shen Q et al. (44) indicated that miR-199a-5p targets the 3’-UTR of DDR1 mRNA to reduce the invasiveness of HCC, and high expression of DDR1 was found to be closely related to advanced HCC and a poor prognosis. Moreover, it is known that the amino acid transporter SLC1A5 and mTORC1 contribute to cellular glutamine transport and glutamine utilization by tumour cells, respectively (65, 66). The DDR1-STAT3 interaction could also be involved in HCC. DDR1 promotes the phosphorylation of STAT3, which in turn increases the DDR1 protein level. The positive feedback between DDR1 and STAT3 promotes HCC tumorigenesis and metastasis to the lung *via* promoting epithelial-mesenchymal transition (EMT) and glutamine metabolism (67). DDR1 also enhances the proliferation of HCC cells *via* the SLC1A5-mediated mTORC1 signalling pathway and induces G1-S phase transition (45). Romayor I et al. (46) demonstrated that inhibition of DDR1 expression or activity suppressed HCC cell adhesion and migration to the ECM in a manner dependent on intercellular adhesion molecule 1 (ICAM1) and vascular cell adhesion molecule 1 (VCAM1) and inhibited HCC cell proliferation, MMP9-dependent degradation of the ECM and the phosphorylation of Akt and extracellular signal-regulated kinase (ERK), which are two pro-survival molecules for tumour cell growth. Transforming growth factor-β1 (TGF-β1) induces collagen crosslinking- and DDR1-mediated linear invadosome formation, which can be reversed by inhibiting DDR1 expression to attenuate the invasiveness of HCC cells (47). Moreover, as a novel ligand of DDR1, the complement protein C1q induces the migration and invasion of HCC cells by activating and upregulating DDR1 and the mitogen-activated protein kinase (MAPK) and PI3K/Akt pathways to increase the expressions of MMP2/9 and EMT-related proteins (48). Concerning DDR2, there is still prominent DDR2 protein expressed on hepatocytes, and inhibiting DDR2 expression can also inhibit the growth, invasion, and migration of HCC cells (49, 50). Furthermore, DDR2 siRNA in HCC cell lines reduces vascular endothelial growth factor (VEGF) expression, suggesting that VEGF may be a downstream target of the DDR2 gene (50).

Early recurrence of liver cancer following surgery is a major prognostic factor. The expression of DDR1 mRNA and protein is prominently higher in early recurrent tumour samples than in nonrecurrent tumour samples, suggesting that DDR1 may be a predictor of early recurrence after hepatectomy (51). Tumour microvascular invasion and circulating tumour cells, which are prognostic factors, are also closely associated with the overexpression of DDR1 (52). Moreover, the serum DDR1 level is significantly higher in HCC patients than in chronic hepatitis patients and healthy individuals (52). After long periods of DDR1 binding to collagen, the N-terminus (extracellular region) of DDR1 undergoes shedding under the induction of a membrane-anchored collagenase and MMPs (68–70). This phenomenon is more pronounced in liver cancer and likely explains the close association between DDR1 in serum and DDR1 in tumours. AFP is perhaps one of the most widely used biomarkers for the evaluation of HCC patient status (71). As more than 50% of HCC patients with low AFP levels or low recurrence risk still have postoperative recurrence and distant metastasis, the monitoring role of AFP faces stern challenges (72). DDR1 still has prognostic value, even in low-risk patients. Thus, the combination of serum DDR1 and tumour DDR1 levels with other monitoring methods, such as alpha fetoprotein (AFP), computed tomography (CT) or magnetic resonance imaging (MRI), might be helpful in forecasting recurrence. Moreover, in a phosphoproteomic screen, DDR1 was identified as a highly phosphorylated RTK in cholangiocarcinoma (73).

### 4.3 Metastatic liver cancer derived from colorectal cancer

Liver metastases and metastatic growth originating from human CRC predict poor prognosis. However, the potential mechanism of DDRs in metastatic liver cancer remains poorly understood. Romayor I et al. (53) showed that metastatic hepatic tissue from CRC expressed high levels of DDR1, which was associated with a poor prognosis. Silencing DDR1 significantly reduced tumour migratory ability and metastatic growth in the liver. The formation of a metastatic focus in the liver is highly dependent on the proliferation of primary tumour cells and the supportive microenvironment (74, 75). Given this information, the interaction between sinusoidal cells (SCs), composed of liver sinusoidal endothelial cells (LSECs), Kupffer cells (KCs) and HSCs, and CRC cells is essential for the metastasis, survival and proliferation of CRC cells in the liver (76). The secretomes of LSECs and HSCs induce increases in the levels of functional DDR1 (phosphorylated DDR1) and MMPs in CRC cells, which further promote CRC cell infiltration into the liver through matrix degradation and ECM remodelling (77). Furthermore, circ-NSD2, a circular noncoding RNA that is highly expressed in liver metastases, upregulates the expression of DDR1, JAG1 (Jagged 1) and their downstream signalling proteins to promote the migration, invasion and metastasis of CRC cells by targeting miR-199b-5p (54).

On the other hand, DDR2 might act as a liver metastasis suppressor through tumour-activated HSCs. We suggest that the most important reason lies in the interaction between DDR2-/- HSCs and cancer cells. Badiola I et al. (55) demonstrated that DDR2-/- HSCs have a higher myofibroblast transdifferentiation rate, release more endothelial cell adhesion molecules and migration-stimulating factors and overexpress key prometastatic factors, including interleukin (IL)-10, VEGF and TGF-β1, in response to CRC cells. However, inhibition of DDR2 signalling in HSCs reduces the expression of MMP2, which is involved in matrix degradation and metastasis promotion (62). Thus, the liver microenvironment produced by DDR2-/- HSC-derived myofibroblasts based on the stimulation of tumour tissue, rather than MMP2 released insufficiently by DDR2-/- HSCs, may be the major contributor to the development of liver metastases derived from colorectal cancer. Interestingly, DDR2 is mainly expressed in HSCs, although it is also seen in hepatocytes or cancer cells (49, 50). This DDR2 expression in different cell types shows functional divergence. This was demonstrated in another study by Badiola I et al. (56), which suggested that downregulation of DDR2 expression in human hepatoma and colon cancer cell lines attenuated their proliferation and migration capacity *in vitro*.

### 4.4 Metastatic liver cancer derived from other cancer types

DDR1 and DDR2 were found to be significantly overexpressed in gastric cancer (GC) cell lines compared to normal gastric mucosal cells, particularly in poorly differentiated GC cells (78, 79). As DDR1 and DDR2 expression increases in GC cells, early recurrence and peritoneal metastasis occur more frequently; thus, we speculate that DDR1 and DDR2 overexpression may also be closely related to further liver metastasis (80, 81). One recent study by Yuge R et al. (57) suggested that DDR1 expression in GC tissue was associated with poor prognosis. By silencing DDR1, GC cell lines were less prone to migration, invasion, and tube formation. DDR1 silencing also reduced GC tissue angiogenesis and lymphangiogenesis in a nude mouse model and almost completely blocked liver colonization and metastasis. Moreover, DDR2 overexpression promotes GC invasion, metastasis and EMT progression by activating mTORC2 and phosphorylating downstream Akt (78). If detected in the early stages of progression, DDR1 and DDR2 may serve as new and promising targets to prevent liver metastasis of GC. This effect of DDRs has also been shown in melanoma. Rather than being expressed in normal tissues, the expression of DDR2 in melanoma cells promotes malignant melanoma growth (82). After intrasplenic inoculation, the liver metastasis ability of DDR2-silenced melanoma cells is reduced compared with that of nonsilenced control cells. The mechanism may be that downregulation of DDR2 in melanoma cells decreases the activity of MMPs to suppress the proliferation, invasion and migration of the cells through inhibition of the ERK1/2 and nuclear factor kappa B (NF-κB) pathways (56).

## 5 DDR inhibitors

The slow development of liver fibrosis associated with a variety of chronic diseases usually takes years (83). There is a slow transition from normal tissue repair to profibrotic tissue and tissue remodelling resulting from a prolonged lesion (84). Early diagnosis and early treatment of liver fibrosis need urgent improvements, and antifibrotic treatment remains stagnant. In addition, the emerging evidence described in this review suggests that DDRs play a crucial role in tumour progression and metastasis. Therefore, this section explains how DDR kinase inhibitors and multikinase inhibitors are used in the treatment of fibrosis and cancer. The effects of multikinase inhibitors may be mediated in part through inhibiting DDRs.

**Figure 4:**
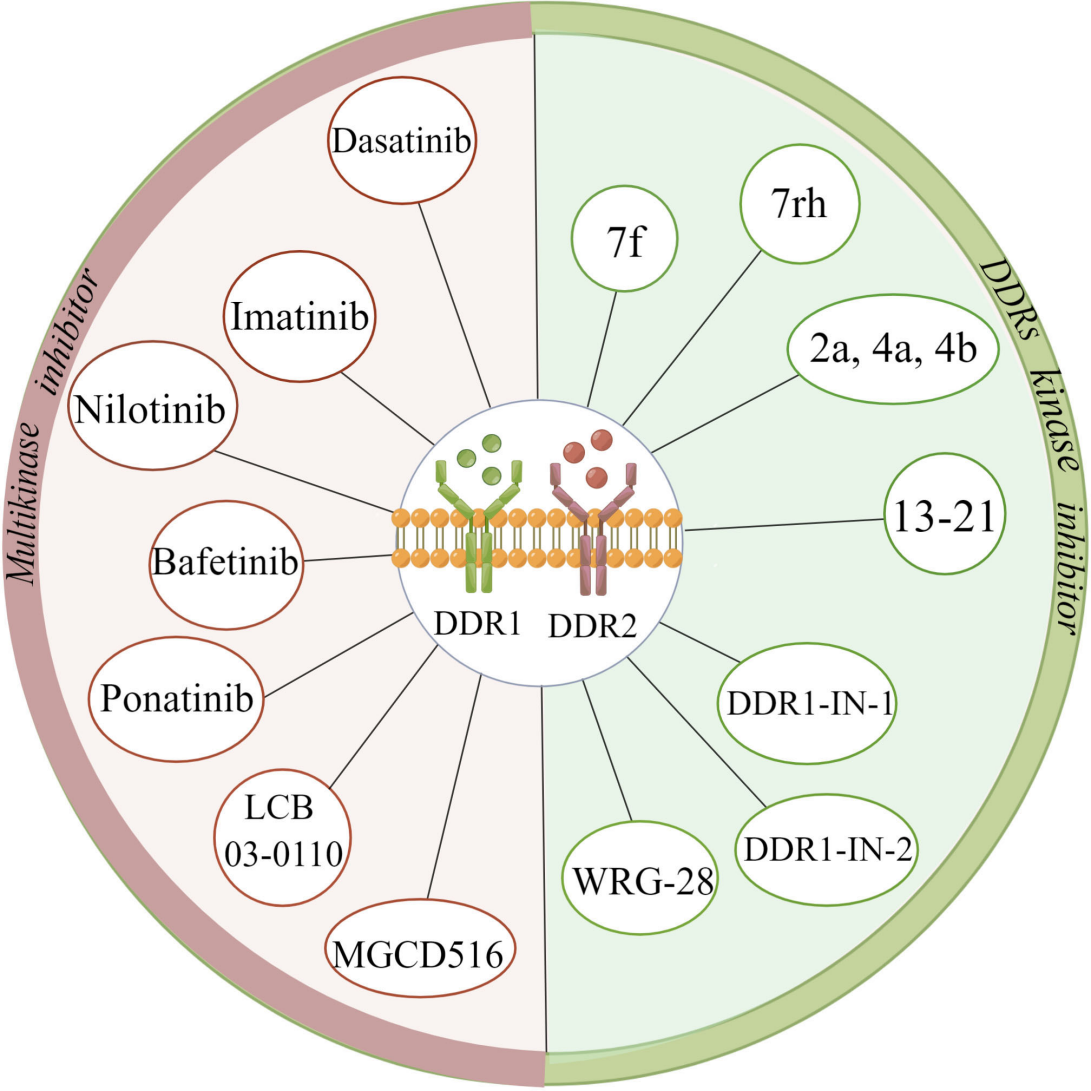
Summary of various discoidin domain receptors inhibitors. (The figure is drawn by Figdraw).

Most DDR inhibitors tend to be adenosine triphosphate (ATP)-competitive inhibitors, which can act on both DDR1 and DDR2 with poor kinase selectivity (85). The multikinase inhibitors dasatinib, imatinib, nilotinib, bafetinib, ponatinib, MGCD516 (sitravatinib) and LCB 03-0110 have been shown to inhibit both DDR1 and DDR2 kinase activity and are mainly applied in the treatment of chronic myeloid leukaemia (CML), lung adenocarcinoma, and colorectal cancer, among other cancer types (86–91). Among them, dasatinib can inhibit the proliferation of squamous cell carcinoma (SQCC) with DDR2 mutation, and ponatinib is prescribed to treat imatinib-resistant patients with CML (88, 92). Concerning DDR kinase inhibitors, the compound 7f is representative of a range of 2-amino-2,3-dihydro-1H-indene-5-carboxamide derivatives; compounds 2a, 4a, and 4b belong to a family of pyrazolourea-containing compounds; and DDR1-IN-1 and DDR1-IN-2 exhibit notable inhibitory effects on DDR1 and DDR2 kinase activity in cancer (93–95). WRG-28 has also been shown to inhibit tumour invasion and metastasis as a selective DDR2 small-molecule inhibitor (96). Moreover, Chen C et al. (97) reported that the novel DDR1 inhibitor AH-487/41940522 can inhibit collagen deposition in a rat model of idiopathic pulmonary fibrosis. Nilotinib has been shown to block the association of myosin IIA with the DDR1 kinase domain to reduce tractional collagen contraction *in vitro* (**Figure 4**) (21). Other potent lead compounds for inhibiting selectively DDRs include compounds 13-21 (98). Among them, compounds 13, 18, and 21 are representative of 1, 2, 3, 4-tetrahydroisoquinoline derivatives, pyrazole fused pyrimidine derivatives, and indazole derivatives against DDR1, respectively; compounds 14-16, compound 17, and compound 19 separately belong to di-amide derivatives, dasatinib analogs and indole analogs against DDR1 and DDR2; and the compound 20 derives from pyridine derivatives against DDR2 (98). Currently, several clinical trials have been conducted with drugs such as dasatinib, nilotinib, and sitravatinib for different cancers, including squamous cell lung carcinoma, advanced or refractory lymphoma, advanced solid malignancies, hematopoietic neoplasm, etc (99–104).

## 6 Drug resistance linked to DDRs

The mechanisms of drug resistance linked to DDRs are associated with DDR mutations. Part of DDR inhibitors loses their inhibitory activity on the background of DDR mutations. The aminopyrimidine head group of imatinib binds to the “gatekeeper” residue Thr701 in DDR1 *via* the hydrogen bond. In CML, the mutation of the “gatekeeper” Thr701 in DDR1 confers steric hindrance and causes resistance to imatinib (105). Ponatinib has been proven to circumvent the steric hindrance and may be selected for the treatment of imatinib-resistant CML patients (86). Considering that Ponatinib is much less selective than imatinib, DDR1-IN-1, therefore, is designed to have a similar pharmacophore model while conferring highly a selective pharmacological profile (94). Beauchamp et al. (106), found that the mutation of the “gatekeeper” T654I in DDR2 and loss of NF1 conferred acquired resistance to dasatinib in lung cancer cell lines *via* targeted exome sequencing. Although the phosphorylation of DDR2 in lung cancer cell lines was decreased by the administration of dasatinib, the acquired mutation in DDR2 T654I blocked this therapeutic effect. And, the loss of NF1 activated a bypass pathway that conferred the activation of the RAS signaling pathway. WRG-28 has been proven to inhibit the collagen-stimulated phosphorylation of DDR2 with T654I mutation *via* its allosteric action upon the extracellular domain (96). Interestingly, a case report by Pitini et al. (107), found a significant reduction in tumor size during administration of dasatinib in one patient with lung squamous SQCC accompanied by S768R mutation of DDR2 kinase gene. However, The high-frequency occurrence of dasatinib toxicity in clinical trials should be noted. Moreover, advanced melanoma with acquired BRAF (V600) mutations is resistant to initially effective BRAF/MEK inhibitors, which is the main reason restricting patient benefit. The key lies in the reactivation of alternate signaling networks, in which the DDR-mediated extracellular matrix is an important component of cancer cell adaptation and resistance to targeted therapy (108, 109). Thus, DDR-targeted therapy can enhance the efficacy of BRAF-targeted therapy to overcome drug resistance.

## 7 Conclusions

Here, we discussed the roles of DDR1 and DDR2 in premalignant liver diseases, primary HCC and metastatic liver cancer derived from several other cancer types. Briefly, the progression of liver fibrosis can be promoted when DDR1 is overexpressed in hepatocytes, and invasion, migration and liver metastasis can be stimulated when DDR1 is overexpressed in tumour cells. However, DDR2 signalling in HSCs can protect against chronic liver fibrosis progression but not early-stage liver injury (prefibrotic stage) and can inhibit liver metastasis of colorectal cancer. These perspectives are critically significant and are first described in detail in this review. Based on relevant original studies, DDR2 can also be expressed in HCC cells and plays a pathogenic role. Although the pathological functions of DDRs in premalignant and malignant liver diseases have been reported, to aid in the development of more targeted DDR inhibitors and facilitate their application in clinical practice, additional studies exploring the concrete molecular mechanisms of DDRs are needed.

## Author contributions

HG: Writing-original draft. H-MX: Designing some contents of the manuscript. D-KZ: Writing, and revising the manuscript. All authors contributed to the article and approved the submitted version.
